# Co-Opetitive Bridging Structure in Rumor Cascades: A Multilayer Overlapping Community Approach with Information-Theoretic Characterization

**DOI:** 10.3390/e28090978

**Published:** 2026-09-02

**Authors:** Sijia Sun, Tian Liu

**Affiliations:** 1 Zhejiang Institute of Administration, Hangzhou 311121, China; 2017101879@ruc.edu.cn; 2 Economic Department, Capital University of Economics and Business, Beijing 100070, China; 3 Leshan Institute of Administration, Leshan 614000, China

**Keywords:** rumor cascade, polarization, overlapping community detection, multilayer network, membership entropy, mutual information, co-opetition, modularity optimization

## Abstract

Rumor events on social media generate opposing camps whose interaction structure is not captured by spreading models or content detectors. This study describes the camp and bridging structure of three rumor events on Sina Weibo, selected from confirmed cases published by the platform’s rumor-refutation channel. Each event is represented as a multilayer interaction network built from repost, comment, and mention relations. Camps are detected by modularity-based community assignment, and overlap is measured through a fractional membership distribution over communities. Three information-theoretic quantities characterize the structure. In the three events, membership entropy separates committed users from bridging users. Structure-to-stance mutual information measures the alignment between interaction communities and text stance. Cross-layer mutual information measures the consistency of camps across interaction types. A co-opetition matrix of mean edge sentiment describes cooperation within camps and competition between camps and identifies alliance structure. In the three events, membership entropy is bimodal, structure-to-stance mutual information is positive and above a permutation null, and the co-opetition matrix has positive diagonal entries. The three events show three distinct temporal patterns, namely a persistent standoff, a hardening toward a single camp after an official correction, and a reversal with an alliance between two camps. The patterns are recovered under a look-ahead-free temporal scheme. In the three events, bridging users hold higher betweenness centrality than non-bridging users. The results describe cross-camp bridging structure in three rumor cascades and connect the structure to a co-opetition reading of camp relations.

## 1. Introduction

Rumors and misinformation on social media affect public trust and can disturb social order during public events [1]. Most quantitative work on rumors follows two lines. The first line models the spread of a rumor with compartmental dynamics and studies control strategies. A recent systematic review reports that susceptible infected recovered models dominate this line and that most studies rely on synthetic data, which limits empirical validation [1]. The second line detects rumor content or rumor sources with machine learning on the propagation graph. Both lines describe how much and how fast a rumor spreads, or whether a message is false. Neither line describes the interaction structure among users, the camps that form around the rumor, or the users who connect these camps.

A separate body of work treats rumor spread as a game between a rumor side and a debunking side. Game-theoretic models study sharing decisions of users [2], adversarial dynamics between rumor spreaders and refuters [3], evolutionary interaction between spreading and refuting behavior [4,5], and reward and punishment mechanisms that involve media, government, and ordinary users [6,7]. These models give a useful account of camp behavior, but they take the camps as given. They do not detect the camps from interaction data, and they do not measure the users who sit between camps.

Research on online polarization studies how users sort into groups with aligned beliefs. Entropy-based methods detect echo chambers and show that a small set of users inside echo chambers can drive a large share of reposts [8]. Signed-network methods describe polarization as antagonism and alignment along a single fault line that splits a population into two opposed camps [9], and negative ties are used to reveal extreme positions [10]. Network-based frameworks quantify the degree of polarization from interaction data [11], and agent-based models study how filter bubbles shape it [12]. Case studies characterize two opposed groups in controversial discourse, for example deniers and believers [13]. Studies of bridging examine whether cross-camp ties survive during partisan conflict [14]. This work makes clear that polarization is not only the presence of two groups. Polarization also concerns the ties that connect the two groups, and the users who hold those ties. Much of this work, however, treats communities as non-overlapping, so the bridging users are not represented as objects of the model.

Community detection offers methods that can represent overlap and multiple relation types. Overlapping community detection in multilayer directed networks addresses the case where a node belongs to more than one community across several directed layers [15]. Overlapping community detection in signed networks addresses positive and negative relations [16], and further methods target overlap in evolving and homophily-driven social graphs [17,18]. Temporal graph methods track how communities in news cascades evolve and reorganize over time [19]. Game-theoretic community detection models community formation as rational choice, where a node joins the same community as friends and a different community from foes [20]. Frameworks that combine community structure with opinion dynamics track how opinions evolve within and across communities [21]. These methods are rarely combined with a stance-alignment test, a co-opetition reading of camp relations, and an event-level dynamic analysis in a rumor setting.

The co-opetition network concept links cooperation and competition to collective outcomes. Cooperation and competition are modeled together as a directed signed graph, and structural balance separates the outcomes into consensus, polarization, and fragmentation [22,23]. This concept is a natural language for rumor camps, because users cooperate within a camp to amplify a shared message and compete across camps through attack and counter-narrative. The concept has not been applied to empirical rumor cascades with detected communities.

This study describes the camp and bridging structure of three confirmed rumor events on Sina Weibo. The three events are selected from cases published by the platform’s rumor-refutation channel, so that the falsity of the rumor and the time of the official correction are documented by the platform. The three events are chosen to cover three types of rumor dynamics identified in prior work: a standoff without an authoritative resolution, a rapid official correction, and a reversal with multiple subcamps. The selection criterion is stated before the analysis, and the measures are then computed in the same way for every event.

This study addresses three research questions, stated at the level of the three events. The first question asks whether the multilayer interaction networks of the three events contain identifiable camps and a separable population of cross-camp bridging users. The second question asks whether, in these events, interactions within camps are cooperative and interactions between camps are competitive and how bridging users are positioned in this signed structure. The third question asks how the measured quantities evolve within each event around its trigger point, and whether the observed patterns persist under a temporal scheme that does not use future information.

The contributions are threefold. First, this study provides an event-level description of camp and bridging structure in three confirmed rumor cascades, with a transparent data construction from the platform’s rumor-refutation channel. Second, this study gives an information-theoretic characterization of bridging and polarization, based on membership entropy, structure-to-stance mutual information, and cross-layer mutual information. Third, this study gives a co-opetition reading of camp relations and bridging roles, using a co-opetition matrix of edge sentiment. The description is supported by four checks: a comparison with baseline community detection methods against platform-documented reference labels, a look-ahead-free temporal scheme, an external betweenness test of bridging users, and a sensitivity analysis of the sentiment labeling rule. This study does not propose a new algorithm, and it does not claim that the three events represent rumor cascades in general. The conclusions are stated for the three events and for the three dynamic types they instantiate.

For researchers studying online conflict, polarization, or rumor governance, this paper provides three reusable components: (1) a transparent pipeline that converts interaction data from a rumor cascade into a camp-and-bridge description; (2) a set of information-theoretic quantities, namely membership entropy, structure-to-stance mutual information, and cross-layer mutual information, that provide a common measurement scale across events of different sizes and topologies; and (3) descriptive evidence from three events that these quantities follow qualitatively different temporal paths, which motivates their use as candidate monitoring signals in platform governance.

The remainder of this paper is organized as follows. Section 2 reviews related work. Section 3 presents the framework, the measures, and the validation protocols. Section 4 describes the data and the experimental setup. Section 5 reports the results. Section 6 discusses the findings. Section 7 states the limitations. Section 8 concludes.

## 2. Related Work

### 2.1. Rumor Spreading and Detection

Compartmental models describe rumor spread through transitions between user states and study control of the resulting dynamics. A systematic review of control-oriented models reports that susceptible infected recovered variants dominate the field and that most studies use synthetic data, which restricts empirical validation [1]. Related work adds community structure or a second layer to the spreading model. A community-based blocking method selects seed nodes for anti-rumor information after a community detection step [24]. A two-layer competitive model studies debunking mechanisms across two layers with time lag effects [25], and work on multilayer diffusion relates intra-layer clustering to how information spreads [26]. Related work identifies the users who play a key role in spreading refutation information [27]. A recent model predicts crucial users in rumor and anti-rumor networks from time-sliced behavioral features with a graph convolutional classifier and tracks how the predicted set changes across slices [28]. These models represent the process of spread and its control or predict influential nodes. They do not analyze the interaction structure of an event as the primary object, and they do not measure the alignment between structure and stance.

### 2.2. Game-Theoretic and Competitive Framings

Game-theoretic models describe rumor and debunking as interacting decisions. A model of sharing decisions treats each user as an agent who acts on a subjective belief and shares news to influence followers and derives the resulting spread as an endogenous outcome [2]. A differential game treats rumor spreading and debunking as interdependent and confrontational behaviors and combines truth dissemination with regulatory measures [7]. An evolutionary game with media, government, and ordinary users classifies posts, comments, and forwards on a microblog platform as rumor or anti-rumor and studies reward and punishment [6]. Further evolutionary-game models describe the interaction between spreading and refuting behavior [4] and correlated information diffusion from a graphical game perspective [5]. A cross-domain framework models competitive interactions between rumor spreaders and refuters with evolutionary game theory [3]. These models motivate the co-opetition reading used in this study. They take the camps as given rather than detecting them, which is the gap the present study addresses.

### 2.3. Polarization, Echo Chambers, and Bridging

Studies of polarization describe how users sort into aligned groups and how cross-group ties change. An entropy-based method detects echo chambers without assumptions about the data and reports that a small fraction of users inside echo chambers produces a large share of reposts [8]. A signed-network method uncovers fault lines and describes polarization as alignment of issues along one axis that splits a population into two antagonistic camps [9]. A case study of controversial discourse characterizes two opposed groups, such as deniers and believers, with social network analysis [13]. A study of bridging examines whether communication between partisan groups survives during conflict [14]. A multiplex analysis of political communities on a microblog platform shows that community patterns differ across layers [29]. A network-based framework quantifies the level of polarization from interaction data with a Louvain step [11], and an agent-based study relates filter bubbles to the emergence of polarization [12]. Work in the Chinese context studies how bystander punishment relates to platform-induced polarization [30]. Opinion-dynamics models describe how competing opinions and social contacts drive polarization and the diffusion of misinformation [31,32]. This work frames polarization as a structural property that includes the bridges between groups. The present study represents these bridges directly through overlapping community membership.

### 2.4. Community Detection: Overlapping, Multilayer, Temporal, and Game-Theoretic

Community detection methods provide the tools for the structural analysis. Modularity optimization by local moving is the standard baseline for weighted undirected networks [33], and map-equation methods detect flow-based communities [34]. Overlapping community detection in multilayer directed networks handles nodes that belong to more than one community across several directed layers and addresses the asymmetric structure of directed layers [15]. Overlapping community detection in signed networks handles positive and negative relations and allows a node to belong to several communities [16]. Label-propagation and seed-extension methods detect overlapping communities in large social graphs [35,36], and mixed membership models represent overlap as a distribution over communities for weighted and bipartite networks [37,38]. Detecting overlap in evolving and homophily-driven graphs has also been studied [17,18]. Temporal multilayer network frameworks model interactions of different types over time [39]. A temporal graph method for fake news cascades learns node embeddings over time, re-detects communities in each snapshot, and models label transitions between snapshots, so that the communities at each time point are estimated from past interactions only [19]. Game-theoretic community detection models the formation of communities as rational choice, with nodes joining friends and separating from foes [20]. A framework that combines a refined community detection with an opinion model tracks the evolution of opinions within and across communities over time [21]. The present study uses modularity-based community assignment with a fractional membership relaxation on a multilayer network and adds an information-theoretic characterization of the overlap together with a stance-alignment test.

### 2.5. Co-Opetition and Structural Balance

The co-opetition network concept models cooperation and competition together. A directed signed graph represents cooperation as positive edges and competition as negative edges, and structural balance separates the collective outcomes into consensus, polarization, and fragmentation [22]. A related study derives conditions for bipartite opinion forming over co-opetition networks [23]. Structural balance partitions a signed graph into subgraphs with positive edges inside and negative edges between subgraphs. Community detection in signed graphs and consensus in signed networks build on the same idea [40,41], and negative ties carry specific information about polarization [10]. This concept matches the co-opetition matrix used in this study, where the diagonal measures cooperation inside camps and the off-diagonal measures competition between camps. The alliance structure observed in one event corresponds to a coalition in the structural-balance sense.

Taken together, the reviewed work provides the components used here. No prior study combines a multilayer overlapping community description, an information-theoretic characterization of bridging and polarization, a co-opetition reading of camp relations, and an event-level dynamic analysis, on confirmed rumor cascades. This combination is the contribution of the present study.

## 3. Framework and Measures

**Figure 1:**
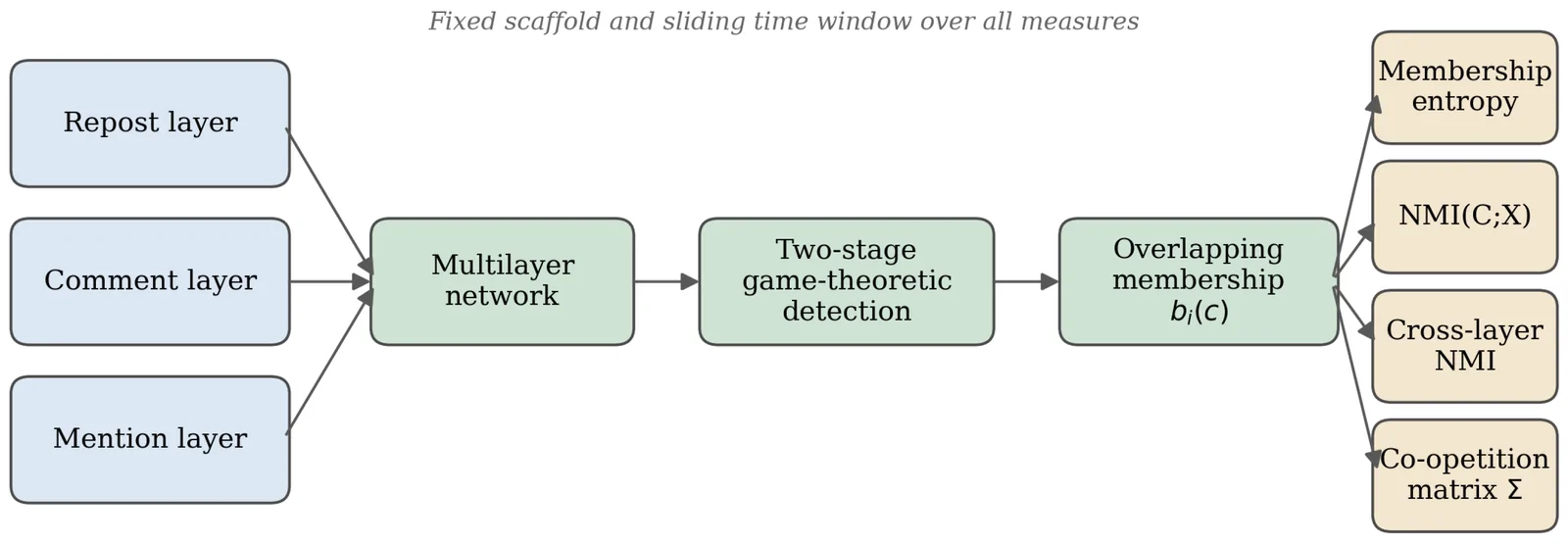
Overview of the analysis pipeline. The three interaction layers, that is repost, comment, and mention, are aggregated into a multilayer network. Modularity-based community assignment returns a hard partition, which is relaxed to a fractional membership distribution over communities. The structure is characterized by membership entropy, structure-to-stance mutual information, cross-layer mutual information, and a co-opetition matrix, and it is tracked over time under three temporal schemes.

Figure 1 gives an overview of the analysis pipeline, from the multilayer interaction network to community assignment, overlap measurement, and the information-theoretic and co-opetition measures.

### 3.1. Multilayer Network

Let V={1,…,N} be the set of users. Let L={rp,cm,mt} be the set of layers, for repost, comment, and mention. For layer *ℓ*, the weighted adjacency $W^{\left(\ell \right)}$ has entry $W_{ij}^{\left(\ell \right)}$ equal to the number of interactions of type *ℓ* from user *i* to user *j* inside the observation window. Community detection uses the symmetrized weight

$$A^{\left(\ell \right)}=\frac{1}{2}\left(W^{\left(\ell \right)}+W^{(\ell )⊤}\right).$$

The multilayer aggregate adjacency is $\bar{A}=\sum_{\ell }\beta_{\ell }A^{\left(\ell \right)}$, with layer weights $\beta_{\ell }\geq 0$ and $\sum_{\ell }\beta_{\ell }=1$. The default uses uniform weights $\beta_{\ell }=1/3$. The layer degree is $d_{i}^{\left(\ell \right)}=\sum_{j}A_{ij}^{\left(\ell \right)}$, the multilayer degree is ${\bar{d}}_{i}=\sum_{\ell }\beta_{\ell }d_{i}^{\left(\ell \right)}$, and the total multilayer weight is $\bar{m}=\frac{1}{2}\sum_{i}{\bar{d}}_{i}$. Nodes are the union across the three layers. A node enters the network if it appears in any layer.

### 3.2. Community Assignment and Overlap

Community assignment uses the multilayer modularity as the objective. Each user *i* receives a single label $c_{i}\in \left\{1,\ldots ,K\right\}$. The assignment is improved by local moves: user *i* adopts the label *c* that maximizes its marginal contribution to the multilayer modularity,

$$u_{i}\left(c\right)=\sum_{\ell }\beta_{\ell }\left(\sum_{j\neq i:\;c_{j}=c}A_{ij}^{\left(\ell \right)}\;-\;\lambda \;\frac{d_{i}^{\left(\ell \right)}\;{\mathrm{vol}}_{\ell }\left(c\right)}{2m_{\ell }}\right),$$

where ${\mathrm{vol}}_{\ell }\left(c\right)=\sum_{j:c_{j}=c}d_{j}^{\left(\ell \right)}$, $m_{\ell }=\frac{1}{2}\sum_{j}d_{j}^{\left(\ell \right)}$, and λ is a resolution parameter. The update $c_{i}\leftarrow \arg\max_{c}u_{i}\left(c\right)$ is the local moving step of modularity optimization [33]. Each local move does not decrease the multilayer modularity

$$Q=\sum_{\ell }\frac{\beta_{\ell }}{2m_{\ell }}\sum_{i,j}\left(A_{ij}^{\left(\ell \right)}-\lambda \frac{d_{i}^{\left(\ell \right)}d_{j}^{\left(\ell \right)}}{2m_{\ell }}\right)\delta \left(c_{i},c_{j}\right),$$

and *Q* is bounded, so asynchronous local moves terminate at a local maximum of *Q*. This update rule can equivalently be written as the best-response dynamic of a non-cooperative game in which $u_{i}$ is the utility of player *i*, and *Q* is an exact potential function, in the sense of game-theoretic community detection [20,22]. The present study uses this equivalence only as a description of the update rule. It does not estimate or test a behavioral game model.

The number of communities *K* is not an input. *K* is the number of communities returned by the local moving procedure at resolution λ on the aggregate graph, and it is determined by the data at the chosen resolution. The value of *K* is reported for each event together with the modularity value, and the robustness of the results to λ is checked in Section 5.9.

The local moving step is repeated from several random initial orders, and the partition with the highest *Q* is retained as the partition $C=\{C_{1},\ldots ,C_{K}\}$.

Overlap is measured by relaxing the hard partition to a fractional membership. For node *i* and community $C_{c}$, the membership coefficient is the fraction of the interaction weight of *i* that points to community $C_{c}$,

$$b_{i}\left(c\right)=\frac{\sum_{\ell }\beta_{\ell }\sum_{j\in C_{c}}A_{ij}^{\left(\ell \right)}}{\sum_{\ell }\beta_{\ell }\sum_{j}A_{ij}^{\left(\ell \right)}},\;\sum_{c}b_{i}\left(c\right)=1.$$

The vector $b_{i}\left(\cdot \right)$ is a probability distribution over communities. It is a descriptive measure of overlap computed from interaction shares; it is not an equilibrium object. With a threshold τ∈(0,0.5], the membership set is $M_{i}=\left\{c:b_{i}\left(c\right)\geq \tau \right\}$. A node with $|M_{i}|\;\geq 2$ is a bridging node. The hard community label is $C\left(i\right)=\arg\max_{c}b_{i}\left(c\right)$. Algorithm 1 gives the procedure as pseudocode, and Table 1 lists all parameter values.

**Algorithm 1** multilayer overlapping camp detection***Input:*** *edge lists of the three layers; λ, τ, $\beta_{\ell }$, restarts R****Output:*** *partition C, membership distributions $b_{i}$, bridging set B*
 *1* 
*Build the symmetrized layer adjacencies $A^{\left(\ell \right)}$ and the aggregate $\bar{A}=\sum_{\ell }\beta_{\ell }A^{\left(\ell \right)}$.*
 *2* 
*Initialize each node with a singleton label.*
 *3* 
*Repeat until no move increases Q: for each node i in a random order, set $c_{i}\leftarrow \arg\max_{c}u_{i}\left(c\right)$.*
 *4* 
*Repeat step 3 from R random orders and keep the partition with the highest Q.*
 *5* 
*Set K to the number of nonempty communities in the retained partition.*
 *6* 
*For each node i and community c, compute $b_{i}\left(c\right)$ from the interaction shares.*
 *7* 
*Set $M_{i}=\left\{c:b_{i}\left(c\right)\geq \tau \right\}$. Mark i as bridging if $|M_{i}|\;\geq 2$. Set $C\left(i\right)=\arg\max_{c}b_{i}\left(c\right)$.*



### 3.3. Information-Theoretic Measures

Membership entropy is the individual-level quantity. For node *i*,

$$H_{i}=-\sum_{c=1}^{K}b_{i}\left(c\right)\log b_{i}\left(c\right),\;{\tilde{H}}_{i}=\frac{H_{i}}{\log K}\in \left[0,1\right].$$

A value near zero indicates a committed user whose interactions are almost all inside one camp. A larger value indicates a user who divides interaction across camps. Entropy defined on network structure has been used to quantify the significance of edges and to identify influential nodes and sources [42,43,44], and the membership entropy used here applies the same idea at the level of community membership. The normalization depends on *K*. For K=2 a fully balanced bridging node reaches one. For K=3 a bridging node across two camps reaches log2/log3≈0.63. The value of *K* is reported for each event, and absolute entropy values are not compared across events with different *K*. Only the shape of the distribution and the trend over time are compared.

The presence of two modes is tested with a comparison between a one-component and a two-component Gaussian mixture, using the Bayesian information criterion. The one-component model is a single Gaussian $N(\mu ,\sigma^{2})$. The two-component model is a mixture of two Gaussians with unconstrained means, variances, and weights. Both models are fitted to the full entropy vector including zero values. The Gaussian mixture does not include an explicit point mass at zero. Support for two modes requires $\Delta \mathrm{BIC}={\mathrm{BIC}}_{1}-{\mathrm{BIC}}_{2}>0$ together with a separation between the two component means. The two-component means, weights, variances, and separation are reported in a supplementary table. The stability of this test is reported through a bootstrap fraction, which is the share of resamples for which the criterion holds. A kernel density estimate provides an independent count of modes. The Sarle bimodality coefficient is reported for description only, together with the coefficient computed after removing the zero-entropy users, to assess whether bimodality is driven by the zero mass. The bimodality claim is stated as a descriptive feature of the entropy distributions rather than evidence for two latent behavioral types. The default bridging threshold τ=0.10 follows the label-retention threshold used in the SLPA family of overlapping community detection algorithms, where r=0.10 is the standard evaluation point [45,46]. A sensitivity analysis reports the bridging fraction at τ=0.05,0.10,0.15,0.20. The bridging fraction inflates at τ=0.05 because minimal cross-camp interaction suffices; at τ∈[0.10,0.20] the fraction stabilizes.

Structure-to-stance mutual information measures the alignment between interaction communities and text stance. Let *C* be the hard community label, and let X∈{support,refute,neutral} be the stance label. With the empirical joint distribution $\hat{p}\left(c,x\right)=n_{cx}/N$,

$$I\left(C;X\right)=\sum_{c,x}\hat{p}\left(c,x\right)\log\frac{\hat{p}\left(c,x\right)}{\hat{p}\left(c\right)\;\hat{p}\left(x\right)},\;\mathrm{NMI}\left(C;X\right)=\frac{I(C;X)}{\sqrt{H(C)\;H(X)}}.$$

A high value indicates that the interaction communities are sorted by stance, which is a signal of polarization. Significance is assessed with a permutation null. The stance labels are shuffled, and the statistic is recomputed for B=1000 resamples, which gives an empirical *p* value. Because the variance of the null is small at the present sample sizes, the *p* value is reported together with the permutation *z* score, which measures the distance of the observed value from the null distribution in units of its standard deviation. This use of mutual information as a stance-alignment measure is consistent with information-theoretic treatments of polarization and echo chambers [8].

Cross-layer consistency measures whether camps are stable across interaction types. The assignment procedure is run on each layer alone, which gives partitions $C^{\left(\ell \right)}$. The pairwise consistency is

$${\mathrm{NMI}}_{\ell ,\ell^{\prime }}=\mathrm{NMI}\left(C^{\left(\ell \right)},C^{\left(\ell^{\prime }\right)}\right).$$

A high value indicates that camps are stable under different interaction types. A low value indicates that the repost camps differ from the comment camps, so that amplification and argument occur among different sets of users.

### 3.4. Co-Opetition Characterization

Each observed edge carries a sentiment sign $s_{ij}\in \left\{+1,-1\right\}$, for endorsement or amplification versus attack or refutation. The definitions follow the co-opetition network reading, where positive edges represent cooperation, and negative edges represent competition [22,23]. The cooperation rate inside camps is $\rho_{\mathrm{in}}^{+}=\mathrm{Pr}\left(s_{ij}=+1|C\left(i\right)=C\left(j\right)\right)$. The competition rate across camps is $\rho_{\mathrm{out}}^{-}=\mathrm{Pr}\left(s_{ij}=-1|C\left(i\right)\neq C\left(j\right)\right)$. The co-opetition matrix is

$$\Sigma_{cc^{\prime }}=\mathrm{mean}\left\{s_{ij}:C\left(i\right)=c,\;C\left(j\right)=c^{\prime }\right\},$$

where the diagonal measures cooperation, and the off-diagonal measures competition. A positive off-diagonal entry between two camps indicates an alliance rather than competition. The set of positive off-diagonal pairs and the set of negative off-diagonal pairs define the alliance structure. A two-camp event has only opposed pairs. A multipolar event can show an alliance, for example a refute camp and an observer camp that tolerate each other while both oppose the rumor core.

Bridging roles describe the position of a bridging node in the signed structure, using the sentiment the node receives from each camp. For bridging node *i* and camp *c*, let $r_{i}\left(c\right)=\mathrm{mean}\left\{s_{ji}:C\left(j\right)=c\right\}$. A node is a mediator if the received sentiment from its main camp is positive. A node is contested if the received sentiment from both sides is negative. A node is a swing node if it receives positive sentiment from one side and negative sentiment from another side. A node is unclassified if it has signed in-edges from fewer than two camps, which is insufficient signal for typing. The role labels are operational definitions of the received-sentiment pattern. They do not by themselves establish that a node mediates information or changes sides.

The payoff-alignment check compares the community assignment with the endorsement each node receives. Let $\pi_{i}\left(c\right)=\sum_{j:C\left(j\right)=c,\;s_{ji}=+1}A_{ji}$ be the positive endorsement that camp *c* gives to *i*. The alignment rate is the share of nodes for which $C\left(i\right)=\arg\max_{c}\pi_{i}\left(c\right)$. A high rate relative to the chance level 1/K indicates that the assigned side matches the camp that gives the most endorsement. The distribution of the own-camp endorsement share $\pi_{i}\left(C\left(i\right)\right)/\sum_{c}\pi_{i}\left(c\right)$ is also reported. Because the edge signs and the stance labels are derived from the same texts, this check is a consistency check between two readings of the data, not an independent test of a behavioral model (Section 7).

### 3.5. Temporal Analysis

The temporal analysis tracks the measured quantities over the lifetime of an event. Three schemes are used, which differ in how the community structure at each window is obtained.

Scheme A, the fixed scaffold, detects the partition $C^{*}$ on the full event and recomputes the membership distributions $b_{i}^{\left(t\right)}$ on the edges of each window (t−W,t], with the scaffold held fixed. Scheme A separates the densification of the network from the dynamics of polarization, but it is an ex post reference frame: the scaffold is estimated from the complete event, so the quantities at early windows are measured relative to a partition that uses later interactions. Scheme A is retained as a descriptive reference frame.

Scheme B, the cumulative scheme, removes the look-ahead. At each window *t*, the partition $C^{\left(t\right)}$ is re-detected on the cumulative edge set of windows 1 to *t*, which contains only interactions observed up to *t*. The tracked quantity is the structure-to-stance mutual information ${\mathrm{NMI}}^{\left(t\right)}\left(C^{\left(t\right)};X^{\left(t\right)}\right)$ between the partition detected at *t* and the stances expressed at *t*. Scheme B is the look-ahead-free check of the temporal patterns.

Scheme C, the independent snapshots scheme, detects the partition separately on the edge set of each single window. Scheme C shows how much of the signal survives when no information is carried across windows. Scheme C returns lower and unstable values with no consistent trigger pattern. This result is substantive: it shows that each contemporaneous window does not independently reveal the event type. The temporal pattern emerges only from the accumulated interaction history. The interpretation is therefore that the cumulative structure changes around the known trigger, not that each snapshot independently signals the event type.

Recent work on fake news cascades estimates the community structure at each time point from past interactions only and models the transitions between snapshots [19]. Scheme B follows the same requirement that the structure at time *t* be a function of interactions observed up to *t*. The object of the temporal analysis here is different: the tracked quantities are event-level distributional signals, namely the mean bridging entropy, the structure-to-stance mutual information, and the concentration of interaction volume, rather than node-level community transitions. The trigger windows are determined from the platform’s event metadata before the temporal analysis is run; they are not inferred from the plotted outcomes. For R2, $t^{*}$ is the window containing the time of the official refutation as recorded by the platform. For R3, $t^{*}$ is the window containing the publicly reported reversal event. Under Scheme B, the partition is re-estimated at each window, and the community labels are not aligned across windows. The tracked quantity, NMI$\left(C^{\left(t\right)};X^{\left(t\right)}\right)$, is invariant to label permutation and therefore does not require label matching. The volume-based quantities (edge-volume entropy and largest camp fraction) are reported only under Scheme A, where the scaffold is fixed and camp identities are constant.

The tracked quantities are the mean bridging entropy over the bridge set *B*,

$${\bar{H}}^{\left(t\right)}=\frac{1}{\left|B\right|}\sum_{i\in B}{\tilde{H}}_{i}^{\left(t\right)},$$

the structure-to-stance mutual information ${\mathrm{NMI}}^{\left(t\right)}$, the edge-volume entropy

$$H_{\mathrm{vol}}^{\left(t\right)}=-\frac{1}{\log K}\sum_{c}\frac{{\mathrm{vol}}^{\left(t\right)}\left(c\right)}{\sum_{c^{\prime }}{\mathrm{vol}}^{\left(t\right)}\left(c^{\prime }\right)}\log\frac{{\mathrm{vol}}^{\left(t\right)}\left(c\right)}{\sum_{c^{\prime }}{\mathrm{vol}}^{\left(t\right)}\left(c^{\prime }\right)},$$

and the largest camp volume fraction $\phi_{\max}^{\left(t\right)}=\max_{c}{\mathrm{vol}}^{\left(t\right)}\left(c\right)/\sum_{c^{\prime }}{\mathrm{vol}}^{\left(t\right)}\left(c^{\prime }\right)$. The edge-volume entropy and the largest camp fraction describe the concentration of interaction toward a single camp, since node membership is close to fixed, and the change appears in interaction volume rather than in node counts. A framework that couples community structure with opinion evolution over time provides related motivation for tracking structure and stance jointly [21].

### 3.6. Validation Protocols

Four checks support the description.

The first check compares the detected structure with baseline methods and with platform-documented reference labels. The baselines are modularity optimization on the aggregate graph [33], a per-layer detection followed by a majority vote across layers, the map-equation method [34], and spectral clustering on the aggregate graph. The reference camp label of a user is derived from the official refutation published by the platform: a user whose expressed stance supports the refuted claim is assigned to the rumor camp, and a user whose expressed stance supports the refutation is assigned to the refute camp. The reference bridging label marks users with observed interactions directed at both camps. Agreement with the reference camps is measured by normalized mutual information, and bridging recovery is measured by precision, recall, and $F_{1}$ against the reference bridging set.

The second check is external to the community structure. If bridging users sit between camps, they should lie on the paths that carry interaction across the network. The betweenness centrality of each user on the repost layer is estimated from 2000 sampled sources. The bridging and non-bridging groups are compared with the rank-biserial correlation *r*, which is the primary effect-size measure. Because the groups are large and the full-sample *p* value is uninformative at this size, the effect size is computed on 50 repeated subsamples of 500 users per group, and the median rank-biserial correlation is reported.

The third check varies the sentiment rule for reposts that carry no added text. Weibo reposts made without comment carry a platform-default text, which is directly identifiable in the data. The default rule assigns such reposts a positive sign, since they amplify the source. The sensitivity analysis recomputes the cooperation rate, the competition rate, and the sign pattern of the co-opetition matrix under two alternative rules, exclusion of these edges and assignment of a negative sign.

The fourth check varies the resolution parameter, the overlap threshold, and the layer weights and repeats the local moving step from several random starts while retaining the partition with the highest modularity.

## 4. Data and Experimental Setup

### 4.1. Platform and Case Selection

The data come from Sina Weibo, a Chinese microblogging platform. Weibo operates a rumor-refutation channel, through which the platform publishes confirmed false claims together with the official refutation. This function provides a documented source of confirmed rumor events: for each published case, the falsity of the claim and the time of the official correction are on record. The platform is chosen for this reason.

Three events are selected from the confirmed cases published by the rumor-refutation channel. The selection criterion is defined before the analysis: the three events should cover three types of rumor dynamics identified in prior work, namely a standoff in which the official refutation does not resolve the dispute, a rapid official correction, and a reversal with multiple subcamps. The type of each event is determined from the event metadata, specifically the timeline of the official refutation and the number of distinct discussion clusters and from preliminary inspection of the discussion threads. Among the confirmed cases, the first event matching each type with sufficient interaction volume is retained. The measures of Section 3 are then computed in the same way for every event. The results are not used to select or to reclassify the events.

Event R1 is a standoff type. It concerns a social controversy in which the official refutation did not resolve the public dispute. Two camps of comparable size persisted throughout the observation window, with frequent cross-camp attacks and a small and persistent set of cross-community users. This type matches the two opposed groups described in studies of controversial discourse [13].

Event R2 is a rapid official correction type. A rumor is corrected quickly by official or media accounts within the first third of the observation window. The event was selected because the platform metadata recorded a clear official correction time, and preliminary inspection of the discussion threads showed a shift in volume after that correction. The measures of Section 3 are computed without reference to the selection metadata.

Event R3 is a reversal and multipolar type. The event was selected because the platform metadata and initial discussion threads showed three or more distinct topic clusters and a publicly reported reversal event within the observation window. The dynamic trajectory is characterized by the analysis, not assumed at the selection stage.

### 4.2. Data Collection

Data were collected from Sina Weibo’s public API. For each event, the seed post was the original rumor post as identified by the platform’s rumor-refutation channel. All repost chains originating from the seed post were followed recursively. Comments were included if they were direct replies to posts in the chain. Mentions were extracted from the text of posts and comments in the chain. Posts deleted before collection are absent from the data. A user can appear in multiple repost chains within the same event; duplicate edges (same source, target, layer, and window) are counted as weight. No filtering for bots or organizational accounts was applied; verified-account status is recorded and available for post hoc analysis. The full recorded lifetime of an event covers the interval from the first observed post to the earlier of (a) two weeks after the official refutation or (b) the time of the last observed repost. The observation periods are R1: 25-11-2022 to 09-12-2022, R2: 14-04-2023 to 28-04-2023, R3: 23-05-2024 to 06-06-2024. For R1, the official refutation was published on 2022-11-25, but the interaction structure shows no convergence after the refutation. The observation window is segmented into *T* equal-width time windows, with T=12 for R1 and R2, and T=14 for R3. No sampling is applied inside an event: all users and interactions recovered from the repost chains enter the data set. Users with no recovered interaction in any layer do not enter the network.

### 4.3. Network Construction

For each event, three directed weighted layers are built from the collected interactions. In the repost layer, a directed edge from *i* to *j* records that *i* reposted a post of *j*, with weight equal to the number of reposts. In the comment layer, a directed edge from *i* to *j* records that *i* commented on a post of *j*, with weight equal to the number of comments. In the mention layer, a directed edge from *i* to *j* records that a post of *i* mentioned *j*, with weight equal to the number of mentions. Edge timestamps are kept for the window analysis. Nodes are the union across the three layers. The sentiment sign of an edge is derived from the interaction text. A repost without added text carries a platform-default text, which is directly identifiable; the default rule assigns such reposts a positive sign, since they amplify the source within the same camp. Alternative rules are evaluated in the sensitivity analysis.

### 4.4. Stance and Sentiment Labeling

Stance and sentiment labels are produced by an automatic procedure with a human check. Two instances of a Qwen 32B model label each item, one with temperature 0.5 and one with temperature 1.0. The inter-model agreement is a Cohen kappa of 0.91. The 200 items for the human check were drawn by stratified random sampling, approximately 67 items per event, with strata proportional to the stance distribution (support: 90; refute: 96; neutral: 14). Two annotators, graduate students with training in public-opinion analysis, independently coded each item following written instructions. The instructions defined support as agreement with, amplification of, or defense of the original claim that was later refuted; refute as disagreement with the original claim, citation of the official refutation, or introduction of counter-evidence; and neutral as discussion without a discernible stance or off-topic content. Annotators coded based on expressed content, not inferred intent, and were provided with three worked examples per category. Disagreements were resolved by a third annotator with domain expertise. The inter-annotator agreement was Cohen’s κ=0.891. The confusion matrix between the automatic labels and the resolved human labels gives an overall accuracy of 93.0%, with class-specific $F_{1}$ of 0.94 for support, 0.95 for refute, and 0.69 for neutral. The neutral category has the lowest $F_{1}$, consistent with its smaller sample size and the ambiguity of uncommitted posts. The stance label takes values in support, refute, and neutral. The edge sentiment sign takes values in positive and negative. A positive edge records a textual interaction with positive sentiment (endorsement; amplification), and a negative edge records a textual interaction with negative sentiment (attack; refutation). The cooperation and competition labels used in the co-opetition characterization are the co-opetition interpretation of these textual signs, not directly observed cooperative or competitive actions. This use of stance labels for rumor analysis follows prior work on stance in rumor settings [47], including joint modeling of rumor veracity and user stance from semantic and structural information [48] and neural stance detection over rumor dialogues [49]. The classification of posts, comments, and forwards as rumor or anti-rumor on a microblog platform follows an earlier study [6].

### 4.5. Reference Labels for Evaluation

The comparison against reference labels uses two platform-documented quantities. The reference camp label of a user is derived from the official refutation: a user whose expressed stance supports the refuted claim is assigned to the rumor camp and a user whose expressed stance supports the refutation is assigned to the refute camp. The reference camp label is a user-level label derived from each user’s dominant stance over the full observation period. It is a binary variable (rumor or refute) for R1 and R2 and a ternary variable for R3. The reference camp *R* and the stance variable *X* are both user-level categorical variables, but they encode different information. The reference camp *R* is binary for R1 and R2 (rumor or refute) and ternary for R3. For R3 the third reference-camp category groups users whose dominant stance over the full event falls outside the rumor–refute axis and whose interactions cluster with a structurally distinct third community; this category is structurally coherent and maps nearly one-to-one to the third detected community. The stance variable X∈{support, refute, neutral} records the aggregated expressed stance of each user. Unlike the third reference-camp category, the neutral stance does not correspond to a single community: neutral users (18.4% in R3; Table 2) are distributed across all three detected communities, which substantially lowers NMI(*C*; *X*). For R1 and R2, where *R* is binary while *X* retains the neutral category, NMI(*C*; *X*) is structurally lower than NMI(*C*; *R*) because the neutral category introduces additional entropy that does not resolve across communities. For R3, two further effects compound the gap: (i) the reversal within the event means that the full-event aggregated stance of users who change sides averages over opposed pre- and post-reversal expressions, so no time-constant user-level stance can align with the partition over the full event, and (ii) the reference camp is constructed to describe which structural side a user belongs to, so high NMI between *C* and *R* is a consistency check on the detection algorithm rather than an independent validation. The aggregated NMI(*C*; *X*) for R3 lies between the pre- and post-reversal window-level alignments. The reference bridging label marks users with observed interactions directed at both camps. The reference labels are used only in the comparison of Section 5.7. They are not used in the detection, the measurement, or the temporal analysis.

### 4.6. Descriptive Statistics

Table 2 reports the descriptive statistics for the three events. The repost layer carries the most edges, followed by the comment layer and then the mention layer, which is consistent with reposts being the main channel of amplification. Verified accounts are a small share of users in all events. The bridging fraction is smallest for R1 and larger for R2 and R3, which is consistent with the standoff type having few and persistent bridges and the reversal type having the most bridges. The stance distribution is close to balanced between support and refute for R1 and R2. For R3 the refute share is substantially larger than the support share (55.4% against 26.2%), and the neutral share is the highest of the three events (18.4%), consistent with a multipolar event in which the reversal shifts many users from support to refute over the observation period.

## 5. Results

Table 3 summarizes the main quantities for the three events. The following subsections report the results by research question.

### 5.1. Camp Structure and the Bridging Population

The detected number of camps matches the event type. R1 and R2 return K=2 camps, and R3 returns K=3 camps, at the default resolution.

**Figure 2:**
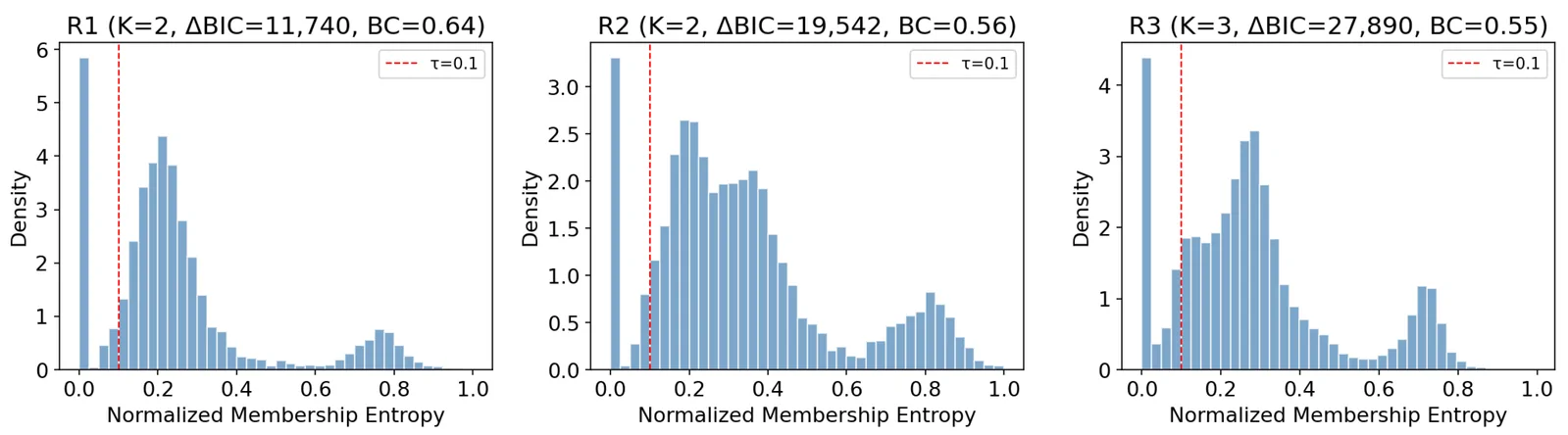
Membership entropy distribution for events R1, R2, and R3. Each panel shows the distribution of the normalized membership entropy over users, together with the two-component Gaussian mixture decision and the bimodality coefficient. The dashed line marks the overlap threshold τ.

The membership entropy distribution has two modes in all three events. A large group of users has entropy near zero, which corresponds to committed users. A second group has higher entropy, which corresponds to bridging users. The two-mode test supports this reading in all events: ΔBIC=11,740 for R1, 19,542 for R2, and 27,890 for R3, each positive and large. The bimodality coefficient is 0.64, 0.56, and 0.55, and it is 0.80, 0.66, and 0.66 after removing the point mass at zero. The values after removing the point mass are not smaller than the raw values, which indicates that the two modes are not an artifact of the committed users at zero. The separation is weakest for R3, which is consistent with the design. R3 has three camps, and for three camps the normalization narrows the gap between the committed mode and the bridging mode. R3 also has the largest bridging fraction, so the region between the two modes is more populated. These results answer the first research question for the three events: the interaction networks contain identifiable camps, and the bridging users form a separable mode in the entropy distribution rather than the tail of a unimodal distribution in these three events (Figure 2).

### 5.2. Structure-to-Stance Alignment

The structure-to-stance mutual information is positive and above the permutation null in all three events. The normalized value, computed from the aggregated user-level stance, is 0.97 for R1, 0.81 for R2, and 0.28 for R3, with an empirical *p* value below 0.001 in all cases from a permutation null with B=1000. The value is highest for the standoff event and lowest for the reversal event. The low value for R3 reflects the reversal: because users change sides during the event, no time-constant user-level stance can align with the structure over the full observation period, and the aggregated value lies between the pre- and post-reversal window-level alignments (see Section 5.6). The observed values lie far outside the null distribution: the null values are concentrated near zero, so the alignment is not explained by the marginal distribution of stances. At the present sample sizes the null variance is small, and even modest alignment would reach conventional significance, so the NMI value itself, rather than the *p* value, is the informative quantity. The use of mutual information and related entropy measures to quantify community structure and its link to political fragmentation and echo chambers has precedent in network studies [11,42]. These results support the reading that, in the three events, interaction communities are sorted by stance.

### 5.3. Co-Opetition Structure and Bridging Roles

**Figure 3:**
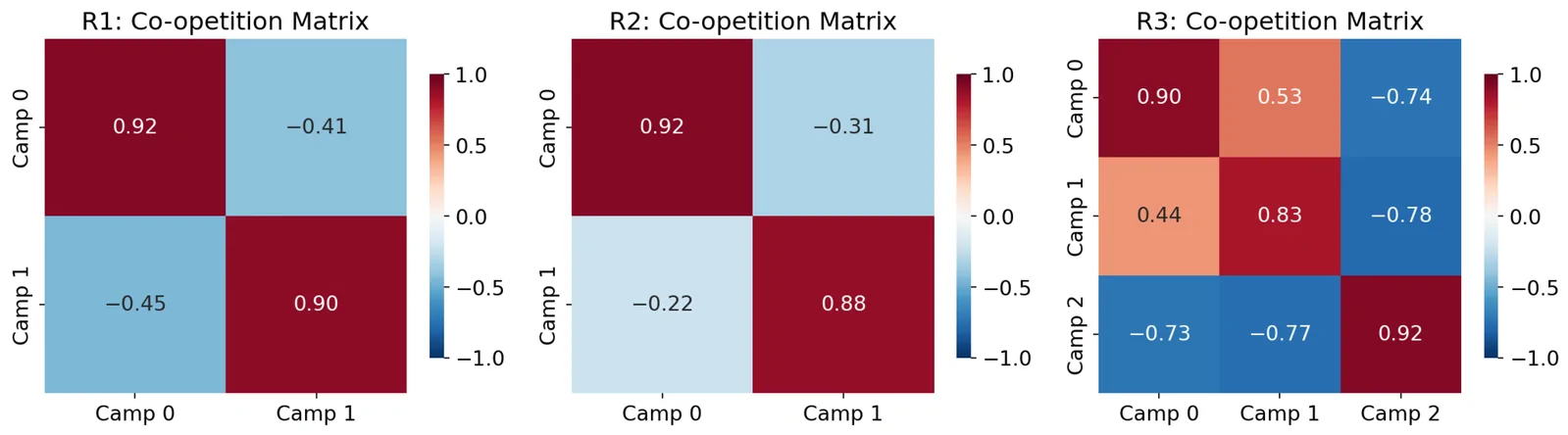
Co-opetition matrix of mean edge sentiment for events R1, R2, and R3. The diagonal gives the mean sentiment of within-camp edges, which measures cooperation. The off-diagonal gives the mean sentiment of between-camp edges, which measures competition. Blue denotes positive sentiment, and red denotes negative sentiment. For R3, the positive off-diagonal entries between two camps indicate an alliance against a third camp.

The co-opetition matrix has positive diagonal entries in all three events, which indicates cooperation within camps. For R1 the diagonal entries are 0.92 and 0.90, and the off-diagonal entries are −0.41 and −0.45, with a cooperation rate of 0.95 and a competition rate of 0.71. For R2 the diagonal entries are 0.92 and 0.88, and the off-diagonal entries are −0.31 and −0.22, with a cooperation rate of 0.95 and a competition rate of 0.62. Both R1 and R2 show two opposed camps with no alliance. For R3 the diagonal entries are 0.90, 0.83, and 0.92. The off-diagonal entries between camp 0 and camp 1 are positive, 0.53 and 0.44, which indicates an alliance. The off-diagonal entries between these two camps and camp 2 are negative, near −0.73 to −0.78, which indicates opposition. The cooperation rate for R3 is 0.94, and the competition rate is 0.42. The lower competition rate for R3 follows from the alliance, which reduces the mean competition across camps. The alliance structure in R3 matches a coalition in the structural-balance sense, in which two camps cooperate against a third [22]. These results answer the second research question for the three events: within-camp interaction is cooperative, between-camp interaction is competitive where the camps are opposed, and the off-diagonal sign pattern identifies an alliance where one exists (Figure 3).

The bridging roles describe the position of bridging users in the signed structure. For R1, among 2151 bridging users, 186 are mediators, 1664 are swing nodes, and 301 are unclassified because they have signed in-edges from fewer than two camps. For R2, among 11,012 bridging users, 2118 are mediators, 6712 are swing nodes, 5 are contested, and 2177 are unclassified. For R3, among 13,670 bridging users, 3453 are mediators, 9088 are swing nodes, 3 are contested, and 1126 are unclassified. The contested role is almost empty in every event. A bridging node sits either inside a friendly region, where it receives positive sentiment from its main camp and is a mediator, or on the fault between opposed camps, where it receives mixed sentiment and is a swing node. The near-absence of contested nodes is consistent with the alliance structure: few bridging users are attacked from all sides.

**Figure 4:**
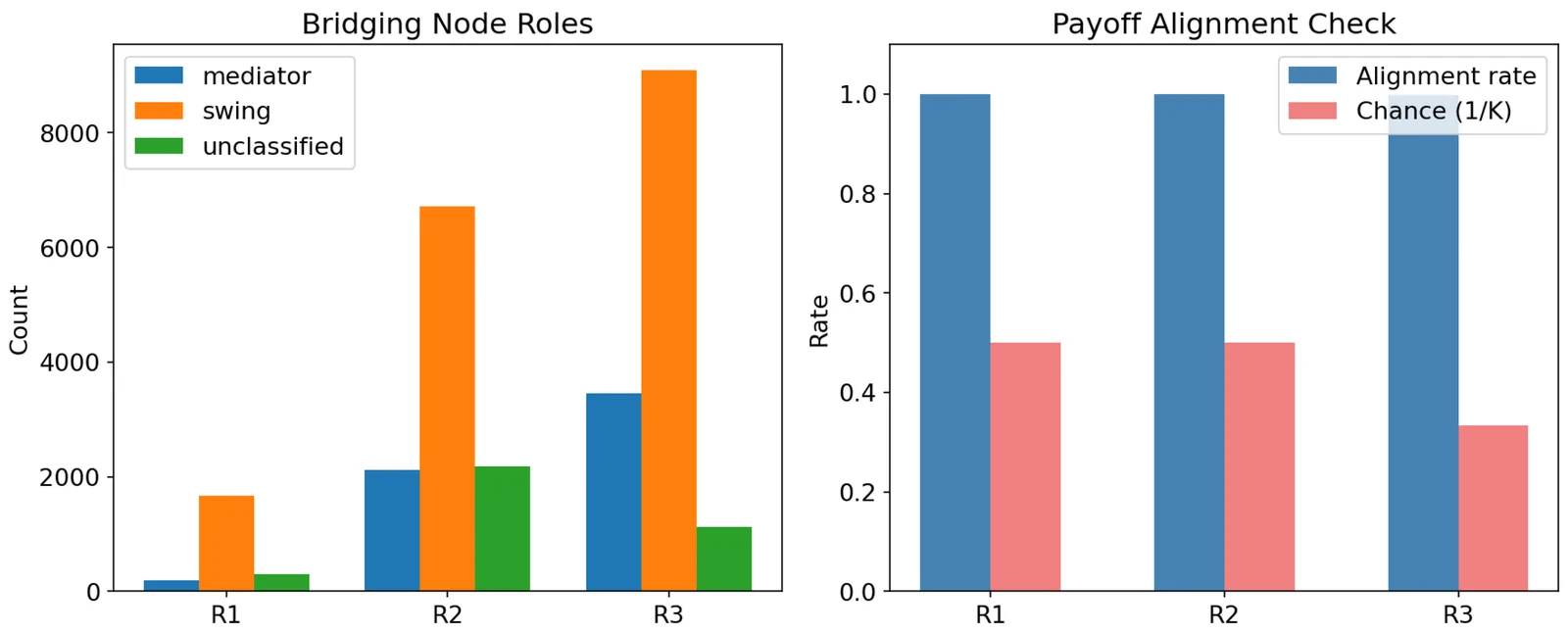
Bridging roles and payoff alignment. The left panel gives the counts of bridging nodes by role, that is mediator, swing, and unclassified, for events R1, R2, and R3. The contested role is omitted because it is almost empty in every event. A node is unclassified when it has signed in-edges from fewer than two camps. The right panel gives the alignment rate against the chance level 1/K, with the median own-camp endorsement share noted below each event.

The payoff-alignment check compares the assigned camp with the endorsement each user receives. The alignment rate is 1.00 for R1 and R2 against a chance level of 0.50 and 1.00 for R3 against a chance level of 0.33. The median own-camp endorsement share is 0.99, 0.98, and 0.94. The assigned side matches the camp that gives the most endorsement for nearly all users. Because the edge signs and the stance labels are derived from the same texts, and because a repost without added text is assigned a positive sign, this correspondence is partly mechanical. The check is reported as a consistency property of the labeling and assignment rules, not as an independent validation of a behavioral model (Section 7). The bridging roles and the own-camp endorsement share are shown in Figure 4.

### 5.4. Temporal Dynamics

The three events show three distinct temporal patterns under the fixed-scaffold scheme (scheme A). The window-level NMI$\left(C;X^{\left(t\right)}\right)$ reported in this section is computed from the stances expressed in each window, which differs from the global NMI(C;X) of Section 5.2, where *X* is the aggregated user-level stance. For R2, the trigger window is $t^{*}=5$, the window of the official correction. Between windows 5 and 6, the window-level structure-to-stance mutual information rises from 0.56 to 0.70, the mean bridging entropy declines in the following windows, the edge-volume entropy declines, and the largest camp fraction rises from 0.51 to a peak of 0.77. This pattern is a hardening of the structure toward a single camp, which matches the rapid official correction type. The rise in the largest camp fraction gives a direct signal of the concentration of interaction toward the refute camp.

For R3, the trigger window is $t^{*}=7$. The structure-to-stance mutual information stays near 0.38 up to window 6 and drops to 0.24 at window 7, while the bridging entropy shows only a mild change, and the edge-volume entropy and the largest camp fraction stay close to flat. This pattern is a reversal. Under a fixed scaffold, the reversal appears as a decoupling of structure and stance, since the fixed communities no longer predict the new stances. It does not appear as a spike in bridging entropy. The flat volume signals indicate that the camps rearrange rather than merge.

**Figure 5:**
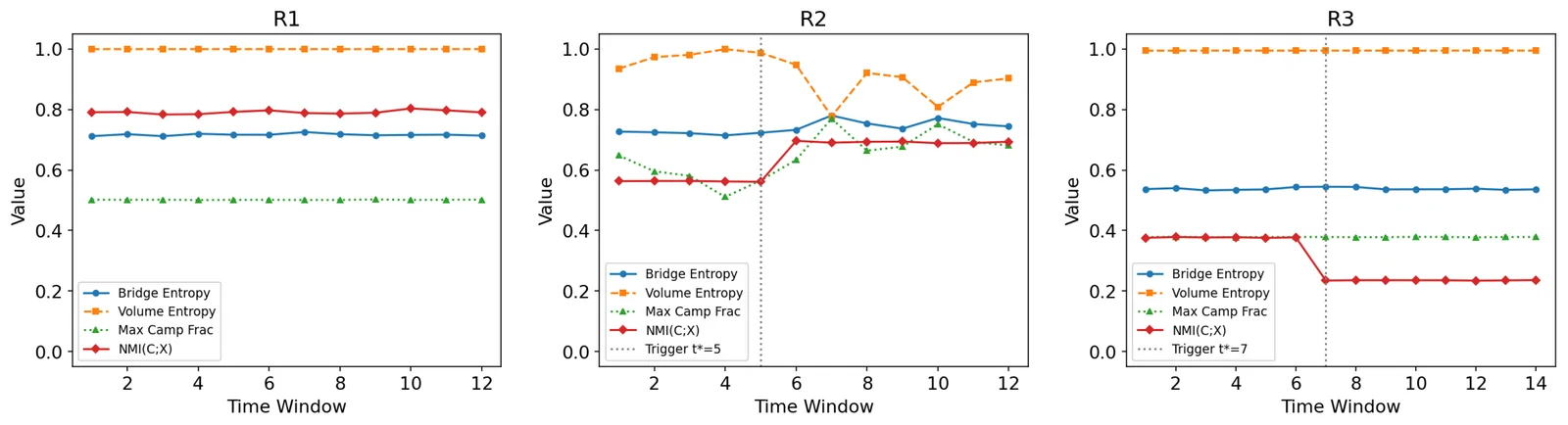
Temporal dynamics for events R1, R2, and R3 under scheme A, the fixed community scaffold. Each panel shows the mean bridging entropy, the edge-volume entropy, the largest camp volume fraction, and the structure-to-stance mutual information across time windows. The dotted line marks the trigger window for R2 and R3. R1 has no single trigger.

For R1, there is no single authoritative trigger, so no trigger window is marked. The structure-to-stance mutual information stays high across the event. The bridging entropy stays within a narrow range and does not collapse. The edge-volume entropy stays high. This pattern is a persistent standoff, which matches the two-camp type. These results answer the third research question at the level of the fixed reference frame: the three events show three distinct temporal patterns (Figure 5).

### 5.5. Cross-Layer Consistency

**Figure 6:**
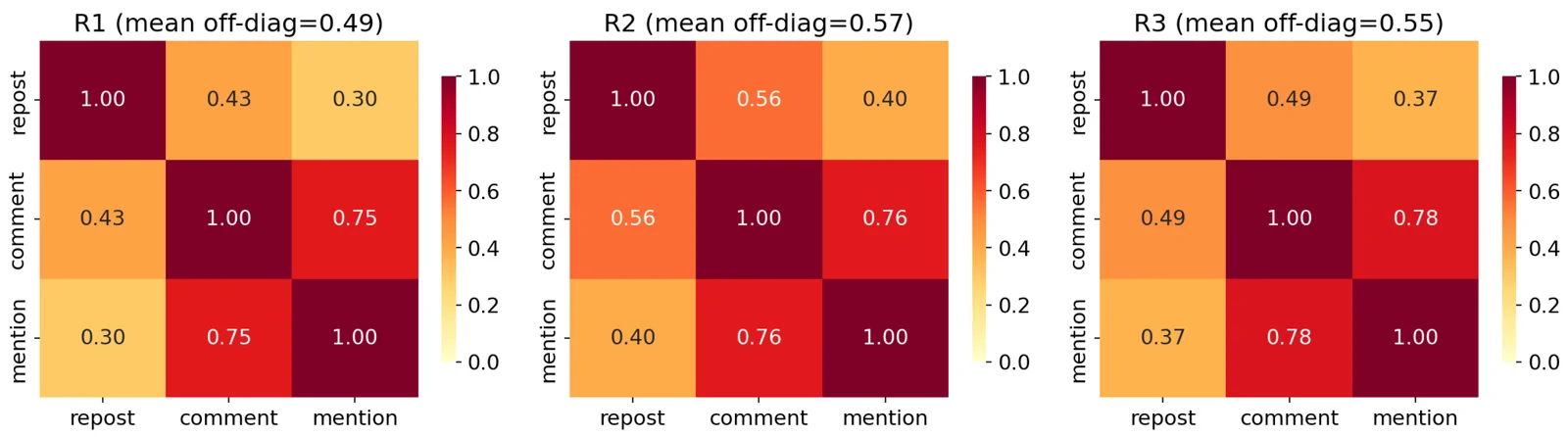
Cross-layer normalized mutual information for events R1, R2, and R3. Each heatmap gives the pairwise normalized mutual information between the single-layer partitions of the repost, comment, and mention layers. Higher values indicate that camps are more consistent across interaction types.

The cross-layer consistency is moderate in all three events. The mean off-diagonal normalized mutual information is 0.49 for R1, 0.57 for R2, and 0.55 for R3. The comment and mention layers are the most consistent pair in every event, with values between 0.75 and 0.78, while the repost layer is the least consistent with the other two. The moderate level in all events indicates that the repost camps, the comment camps, and the mention camps are only partly consistent, which supports the use of separate layers rather than a single merged graph. A multiplex analysis of political communities reports a similar finding that community patterns differ across layers [29]. The interpretation of the structure-to-stance results therefore rests on the aggregate partition, which is the object that carries the high mutual information with stance. The pairwise cross-layer values are shown in Figure 6.

### 5.6. Temporal Analysis Without Look-Ahead

**Figure 7:**
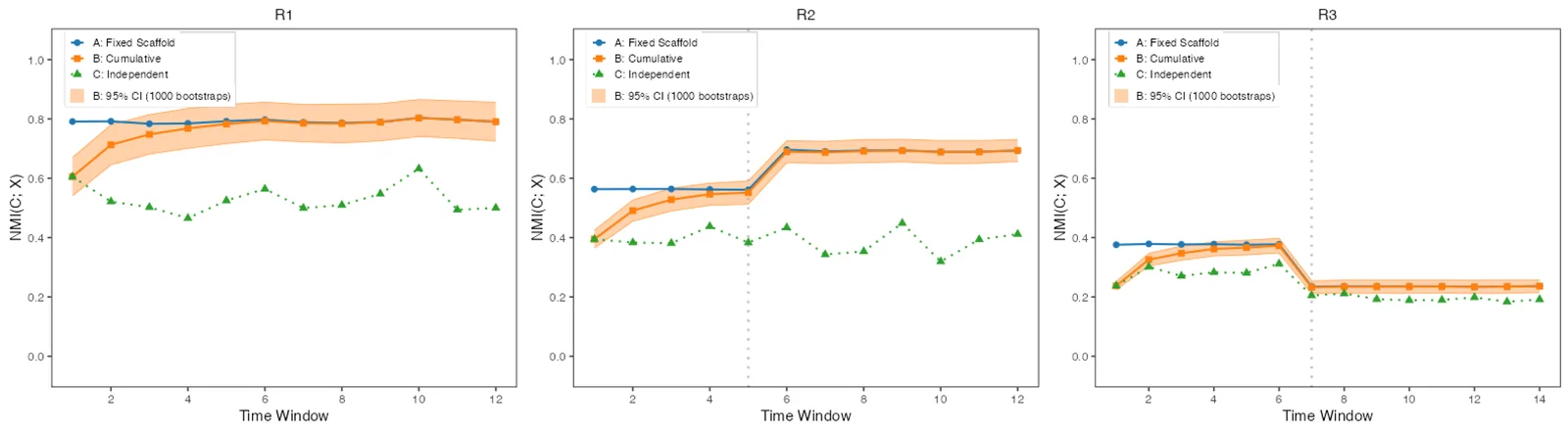
Structure-to-stance mutual information over time under three temporal schemes. Scheme A uses the fixed full-event scaffold. Scheme B re-detects the partition on the cumulative interactions up to each window and uses no future information. The shaded band gives the 95% conditional bootstrap band for scheme B, computed from 1000 resamples of the users at each window with the partition held fixed. Scheme C re-detects the partition on each window independently. The dotted line marks the trigger window for R2 and R3.

Scheme A measures early windows relative to a partition estimated from the complete event. Scheme B removes this look-ahead: at each window *t*, the partition is re-detected on the cumulative interactions observed up to *t*. Figure 7 compares the structure-to-stance mutual information under the three schemes.

The trigger patterns of Section 5.4 persist under scheme B. For R2, the cumulative NMI rises from 0.55 at window 5 to 0.69 at window 6 and stays near 0.69, the same hardening step seen under scheme A. For R3, the cumulative NMI is near 0.37 up to window 6 and drops to 0.23 at window 7, the same reversal step. For R1, the cumulative NMI starts below the scaffold value and converges to it as the network densifies, with no trigger. The three temporal patterns therefore do not depend on the ex post scaffold: they appear when the structure at time *t* is estimated from interactions observed up to *t* only. The bootstrap bands shown in Figure 7 hold the estimated partition fixed and resample users independently. They describe the variability of the NMI statistic conditional on the partition and do not account for partition uncertainty or dependence among users who share edges. They are conditional descriptive bands, not a complete assessment of sampling uncertainty.

Scheme C, which re-detects communities on each window alone, returns lower and unstable values, between 0.18 and 0.63 across events and windows, with no consistent trigger pattern. Single windows are sparse, so independent detection fragments the structure. The comparison shows why a cumulative design is needed: the signal is carried by the accumulated interaction history, not by any single window.

This design follows the requirement that the community structure at time *t* be a function of interactions observed up to *t*, which is the requirement met by temporal graph methods for fake news cascades [19]. The tracked object here is different: an event-level alignment signal rather than node-level community transitions.

### 5.7. Comparison with Baseline Methods

Table 4 compares the detected structure with baseline methods against the platform-documented reference labels. The NMI(C;X) column reports the structure-to-stance mutual information computed from the aggregated user-level stance. The first stage of the proposed procedure is the local moving step of modularity optimization on the aggregate graph, so the proposed method, its hard-partition variant, and the Louvain-Aggregate baseline target the same objective on the same graph. Because the optimization is a heuristic that depends on the processing order of nodes, different runs converge to different local optima. In every event the three resulting partitions are near-identical: NMI(C;X) and NMI against the reference camps agree to three decimal places. The *Q* values differ slightly (within 0.02 in every event), because *Q* is evaluated on each method’s own partition and is more sensitive to individual node reassignments than the normalized mutual information is. The comparison therefore separates two questions: whether the aggregate partition recovers the reference camps and whether a method can represent cross-community users.

On the first question, the aggregate partition recovers the reference camps in all events, with NMI against the reference ranging between 0.99 and 1.00 for every modularity-based method. Infomap returns many small communities (K=12, 66, and 39 against 2, 2, and 3), which fragments the camps while keeping a high NMI. The majority vote across single-layer partitions returns comparable values, which shows that the aggregate graph does not lose camp information relative to per-layer detection.

**Figure 8:**
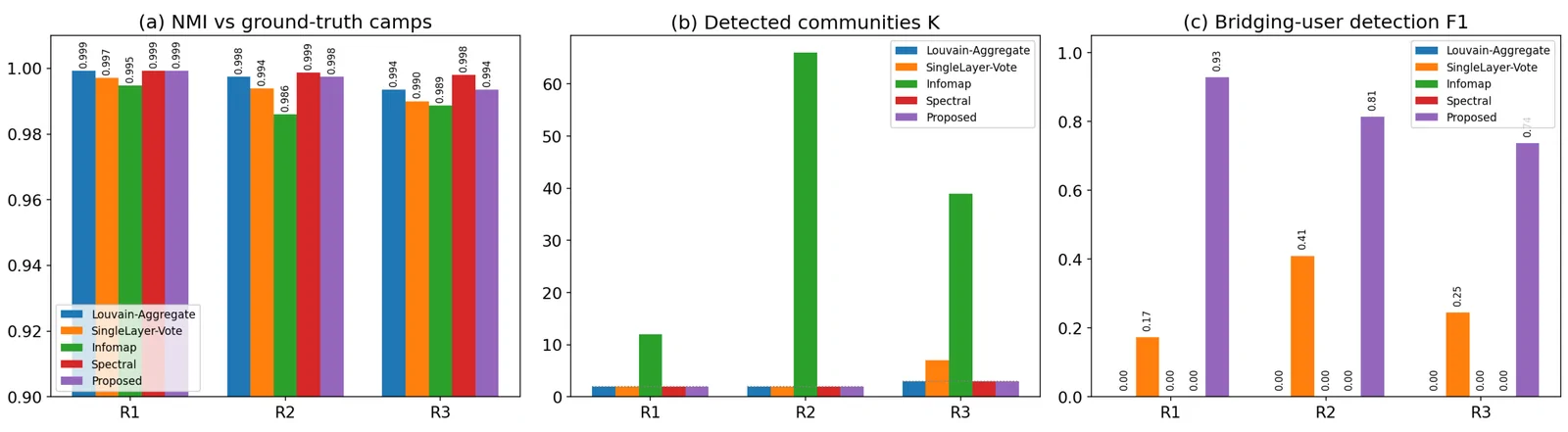
Comparison with baseline methods. (**a**) NMI against the reference camps; all modularity-based methods recover the camps, and the axis is truncated for readability. (**b**) Detected number of communities *K*; Infomap over-segments in every event. (**c**) Bridging-user detection F1 against the reference bridging set; hard-partition methods have no overlap representation and score zero by construction.

On the second question, hard-partition methods have no representation of overlap, so their bridging precision, recall, and $F_{1}$ are zero by construction. These entries describe representational capability, not detection accuracy. The SingleLayer-Vote baseline marks nodes whose layer labels disagree as bridge-like and recovers a fraction of the reference bridges, with $F_{1}$ between 0.17 and 0.41. The fractional membership of the proposed procedure recovers the reference bridges with $F_{1}=0.93$ for R1, 0.81 for R2, and 0.74 for R3, at the same partition quality as the hard methods. The comparison indicates that the value of the proposed combination lies in the overlap layer: it adds a bridging representation to a modularity partition at no cost to partition quality (Figure 8).

### 5.8. Bridging Users and Betweenness Centrality

**Figure 9:**
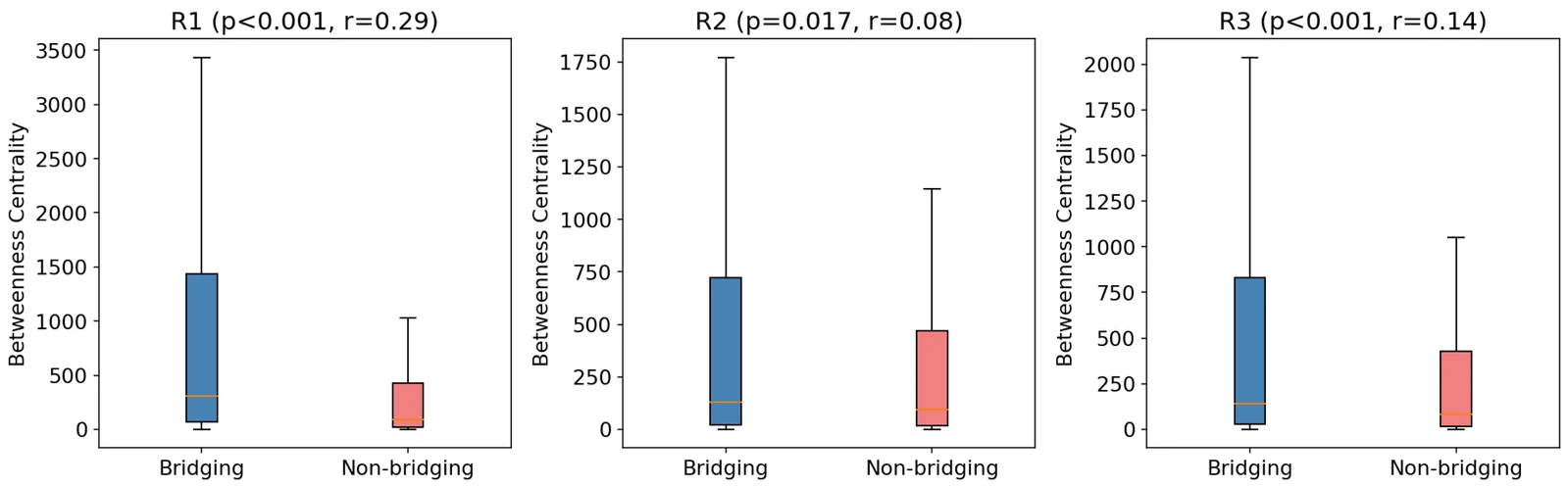
Betweenness centrality of bridging and non-bridging users on the repost layer. Each panel gives the distribution for the two groups and the rank-biserial correlation *r*.

The betweenness check tests whether bridging users occupy positions that carry interaction across the network, a property that is external to the membership coefficients used to define them. Table 5 reports the comparison on the repost layer. In all three events, the median betweenness centrality of bridging users is higher than that of non-bridging users. The rank-biserial correlation is r=0.29 for R1 (median betweenness 307.2 against 92.3), r=0.08 for R2 (129.6 against 94.2), and r=0.14 for R3 (141.6 against 81.6), indicating a small-to-moderate effect that varies by event type. The small effect in R2 is consistent with the event type: after the official correction, interaction concentrates on the correction hub, and cross-camp paths are short, so few users sit on bottlenecks. The check supports the structural reading of the bridging label (Figure 9).

### 5.9. Robustness and Sensitivity

The permutation null for the structure-to-stance mutual information gives an empirical *p* value below 0.001 in all three events. The analysis also varies the resolution parameter, the overlap threshold, and the layer weights, and it repeats the local moving step from several random starts while retaining the partition with the highest modularity. Across these variations, the main qualitative conclusions remain in place. The membership entropy stays bimodal, the structure-to-stance mutual information stays positive and significant, the sign structure of the co-opetition matrix stays the same, and the three temporal patterns stay distinct. The conclusions therefore do not depend on a single parameter choice.

**Figure 10:**
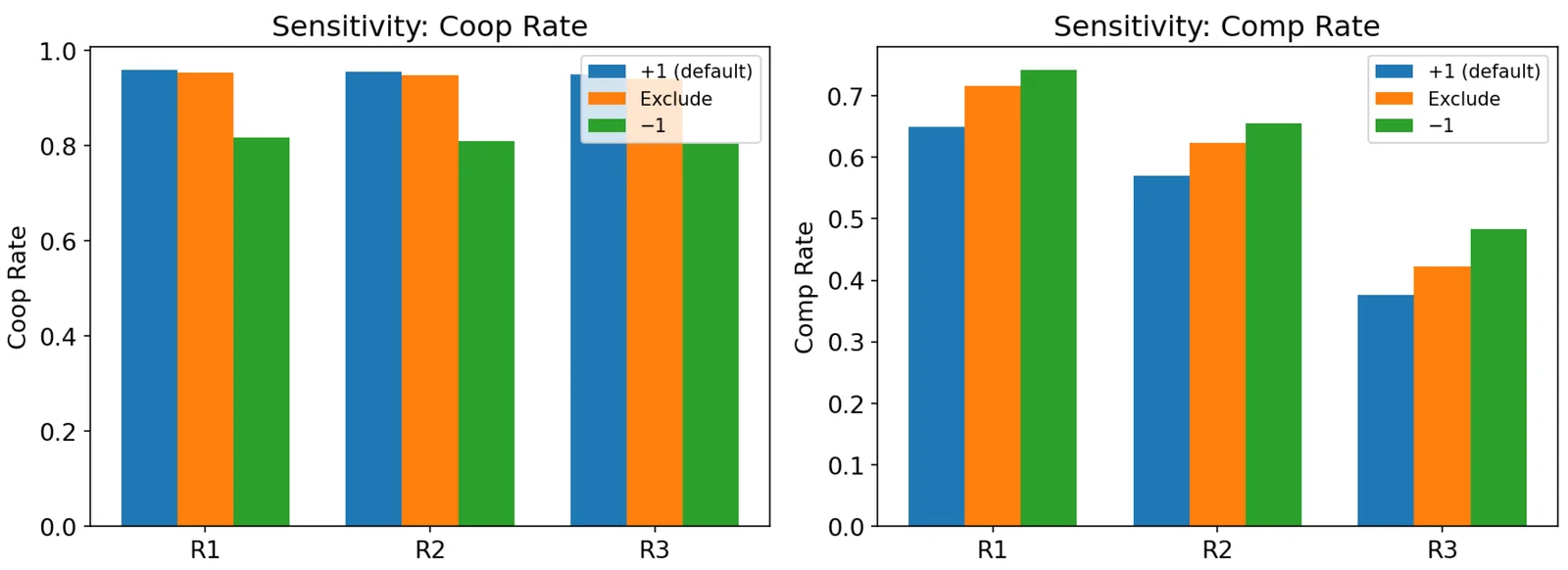
Sensitivity of the cooperation and competition rates to the sentiment rule for reposts without added text. The three rules assign such reposts a positive sign, exclude them, or assign a negative sign.

The sentiment default rule for reposts without added text is varied separately. Table 6 reports the cooperation rate, the competition rate, and the sign pattern of the co-opetition matrix under three rules: the default positive assignment, the exclusion of these edges, and a negative assignment. The diagonal of the co-opetition matrix stays positive under all three rules in all events. The cooperation rate declines when no-text reposts are excluded or signed negative, which is expected because these edges are predominantly inside camps; the competition rate moves in the opposite direction. The qualitative conclusion, cooperation within camps and competition between opposed camps, does not depend on the default rule (Figure 10).

## 6. Discussion

The measures describe three distinct patterns in the three events. The pattern differences are carried most strongly by the structure-to-stance mutual information over time and by the co-opetition matrix. Table 4 confirms that the hard partition returned by the proposed procedure is near-identical to that of aggregate Louvain (NMI(C;X) and NMI against the reference agree to three decimal places; the *Q* values differ within 0.02 because the Louvain heuristic can converge to different local optima across runs). The distinctive contribution is the second-stage fractional membership post-processing and the subsequent information-theoretic and co-opetition descriptive statistics, not a new community-formation mechanism. The standoff event stays polarized with a high and stable alignment. The rapid correction event hardens toward a single camp after the trigger. The reversal event shows a sharp drop in alignment at the trigger together with an alliance among camps. The same three patterns appear under the cumulative scheme that uses no future information, so the separation is not an artifact of the fixed reference frame.

The findings connect to the co-opetition network reading of collective outcomes. Cooperation and competition on a signed graph separate into consensus, polarization, and fragmentation through structural balance [22,23]. The three events map onto these outcomes. R1 corresponds to polarization, since two camps oppose each other with a stable structure. R2 corresponds to a move toward consensus, since interaction concentrates on a single camp after the correction. R3 corresponds to fragmentation with a coalition, since three camps form and two of them ally against the third. The alliance in R3 is a coalition in the structural-balance sense, and it explains the lower competition rate for that event.

The findings also connect to signed-network and information-theoretic studies of polarization. The co-opetition matrix is a community-level reading of alignment and antagonism, which is the property that fault-line methods measure at the level of nodes and edges [9]. The bridging users are a structural minority whose state tracks the state of the event, which parallels the finding that a small set of echo-chamber users drives a large share of reposts [8]. The high-activity correction hub in the rapid correction event is consistent with data-driven accounts of online influence, in which a small set of accounts carries a large share of the interaction [51]. The decline of bridging entropy signals hardening, and the drop in structure-to-stance mutual information signals reversal. These two signals give distinct indicators for the two dynamics.

The temporal analysis complements temporal graph methods for fake news cascades. Ma, Qu, and Wang re-detect communities at each snapshot from past interactions and model node-level label transitions, which shows that communities in fake news cascades evolve and reorganize over time [19]. The present analysis makes the same methodological requirement, that the structure at time *t* use only interactions observed up to *t*, and applies it to a different object: event-level signals, namely the bridging entropy, the structure-to-stance alignment, and the concentration of interaction volume. The two objects answer different questions. Node-level transitions describe how communities reorganize; the event-level signals describe which type the event belongs to. A combination of the two, tracking community lifecycles and camp alliances jointly, is a natural extension.

The cross-community users are also distinct from the crucial users studied in rumor and anti-rumor diffusion. Wang et al. predict the users who will be influential in the next time slice from behavioral features, a supervised importance-ranking task [28]. The bridging users identified here are defined by structure alone: they are the users whose interaction weight is divided across camps. A low-degree user can be bridging without being influential, and a high-degree inside-camp account can be influential without bridging. The betweenness check gives external support to the structural reading: bridging users lie on the paths that carry reposts, with an effect size that depends on the event type. The role labels used here, mediator and swing node, are operational labels for the received-sentiment pattern and do not by themselves establish that a node mediates information flow or changes sides.

The payoff-alignment result has a narrower interpretation than in a behavioral reading. The assigned camp matches the camp that gives the most endorsement for nearly all users, but the endorsement signs and the stance labels are derived from the same texts, and a repost without added text is assigned a positive sign. The near-ceiling alignment is therefore a consistency property of the construction, not evidence that users choose sides by maximizing endorsement. We report it because it documents how the labeling and assignment rules interact, and because it sets the scale against which any future independent test of side choice must be read.

The findings have implications for platform governance, stated at the level of the three events. The cross-community users are the nodes that connect camps, so their state is a useful monitoring target. A decline in bridging entropy describes hardening toward a single camp, and a drop in structure-to-stance mutual information describes a reversal of stance. A platform could in principle track these two descriptive temporal signals to distinguish a hardening event from a reversal event, pending validation on a larger and prospectively sampled set of events. The co-opetition matrix can reveal an alliance among camps, which indicates that the conflict is not a simple two-way opposition. Whether these descriptive signals can serve prospectively as early indicators for intervention requires testing on a larger set of events without using subsequent outcomes to select or label them.

## 7. Limitations

The analysis has several limitations. First, the three events are purposively selected to instantiate three dynamic types, and the analysis is descriptive. The results characterize these three events and the types they represent; they do not support quantitative claims about rumor cascades in general, such as the frequency of alliances or the base rate of bridging. Second, the detected structure is an interaction structure, not a stance structure. The camp reading rests on the alignment between structure and stance, which is measured by the structure-to-stance mutual information. This alignment step is required before the reading is applied, and the design is associational, not causal: the hardening and the reversal are observed patterns of co-variation, and the mechanism reading is an interpretation. Third, the stance and sentiment labels carry uncertainty. The labels are produced by an automatic procedure with a human check, and the agreement is reported as a Cohen kappa of 0.91 between models and 0.87 against a human sample. Label error reduces the measured mutual information, so the reported values are conservative. Fourth, the stance labels and the edge sentiment signs are derived from the same texts. The payoff-alignment check is therefore partly mechanical and is reported as a consistency property, not as independent evidence for a behavioral account of side choice. Fifth, the fixed-scaffold temporal scheme is an ex post reference frame. The cumulative scheme removes the look-ahead and recovers the same patterns, but re-detection on cumulative data introduces its own variability, and the independent-snapshot scheme shows that single windows are too sparse to carry the signal. The temporal conclusions are stated for the quantities that behave consistently across the two usable schemes. Sixth, the reference labels used in the baseline comparison are derived from the official refutation and from observed cross-camp interaction. For R3, the third reference-camp category groups users whose dominant stance falls outside the rumor–refute axis and whose interactions cluster with a distinct third community; this category is structurally coherent and maps nearly one-to-one to a detected community. The comparison therefore measures agreement with a structurally aligned reference, not with an independently annotated ground truth, and the high NMI(*C*; *R*) is partly a consistency property of the construction. The harder alignment test is NMI(*C*; *X*), which uses the raw three-category stance and returns a lower value (0.28 for R3), because the neutral category is dispersed across communities, and the reversal weakens the full-event aggregation. Seventh, the betweenness effect is small for the correction event, so the external support for the bridging label is uneven across event types. Eighth, the membership entropy normalization depends on the number of camps, so absolute entropy values are not compared across events. Ninth, the cross-layer consistency is moderate in all events and does not separate the event types, so this quantity is a secondary and descriptive result in this study.

## 8. Conclusions

This study described three confirmed rumor events on Sina Weibo as multilayer interaction networks. Camps were detected by modularity-based community assignment, and overlap was measured through a fractional membership distribution over communities. Three information-theoretic quantities characterized the structure, namely membership entropy, structure-to-stance mutual information, and cross-layer mutual information. A co-opetition matrix of edge sentiment described cooperation within camps and competition between camps and identified alliance structure. In the three events, membership entropy was bimodal, structure-to-stance mutual information was positive and above a permutation null, and the co-opetition matrix had positive diagonal entries. The three events showed three distinct temporal patterns, namely a persistent standoff, a hardening toward a single camp after an official correction, and a reversal with an alliance among camps, and the patterns persisted under a look-ahead-free temporal scheme. Cross-community users held higher betweenness centrality than non-bridging users, and the co-opetition conclusions were stable under the sentiment labeling rule.

Concretely, the reader can take away four operational results. First, the membership-entropy bimodality test provides, in the three events studied, a diagnostic for whether the entropy distribution contains a separable bridging mode, without supervised labeling. Second, the NMI(*C*; *X*) trajectory under cumulative analysis distinguishes, in these three cases, standoff (flat), hardening (rising), and reversal (falling) dynamics; the same computation applies to any event with timestamped interaction data. Third, the co-opetition matrix Σ gives a compact, size-independent representation of sentiment flow between and within camps that can be compared across events. Fourth, the threshold-sensitivity analysis of τ offers a template for reporting the robustness of overlap-based measures in future studies. Researchers can apply the same pipeline to their own rumor or polarization data to obtain comparable descriptions.

The description is limited to the three events, and the results characterize the three dynamic types they instantiate. Establishing whether the proposed measures can prospectively classify an unknown event requires applying them blind to a larger set of events without using subsequent outcomes to select or label them. Future work can extend the analysis to a larger set of events, can add external validation of cross-community users through account attributes and manual annotation, can combine event-level signals with explicit tracking of community lifecycles, and can test the two descriptive signals as prospective indicators for intervention.

## Figures and Tables

**Table 1:** Parameter values used in the analysis.

Parameter	Meaning	Value
λ	resolution of the multilayer modularity	1.0
τ	overlap threshold for the membership set	0.1
$\beta_{\ell }$	layer weights	1/3 each
*R*	random restarts of local moving	10
*T*	time windows per event	12 (R1, R2), 14 (R3)
*B*	permutation resamples for NMI significance	1000
*s*	source samples for betweenness estimation	2000
$n_{\mathrm{sub}}\times r$	subsample size and repetitions for the Mann-Whitney check	500 × 50

**Table 2:** Descriptive statistics of the three events.

Quantity	R1	R2	R3
Users (*N*)	18,032	55,479	70,017
Time windows (*T*)	12	12	14
Repost edges	886,552	2,623,803	3,545,997
Comment edges	691,274	2,075,710	2,794,309
Mention edges	326,201	904,007	1,269,813
Total edges	1,904,027	5,603,520	7,610,119
Verified accounts (%)	0.6	0.5	0.6
Bridging nodes (%)	11.9	19.8	19.5
Stance: support (%)	49.9	49.5	26.2
Stance: refute (%)	50.0	49.2	55.4
Stance: neutral (%)	0.1	1.3	18.4

**Table 3:** Summary of the main structural, co-opetition, and dynamic quantities. NMI(C;X) is the structure-to-stance normalized mutual information, with permutation p<0.001 for all events. Cross-layer NMI is the mean off-diagonal value.

Quantity	R1	R2	R3
Number of camps *K*	2	2	3
Bimodality ΔBIC	11,740	19,542	27,890
Bimodality coefficient BC	0.64	0.56	0.55
NMI(C;X)	0.97	0.81	0.28
Cooperation rate $\rho_{\mathrm{in}}^{+}$	0.95	0.95	0.94
Competition rate $\rho_{\mathrm{out}}^{-}$	0.71	0.62	0.42
Cross-layer NMI (mean off-diagonal)	0.49	0.57	0.55
Dynamic type	standoff	hardening	reversal
Trigger window $t^{*}$	none	5	7

**Table 4:** Comparison with baseline methods against the platform-documented reference labels. NMI(C;X) is the structure-to-stance mutual information (computed from the aggregated user-level stance), *Q* is the multilayer modularity evaluated on each method’s own partition, NMI(ref) is the agreement with the reference camps, and bridge P, R, and F1 measure recovery of the reference bridging set. Hard-partition methods (marked †) have no overlap representation, so their bridging scores are zero by construction. SLPA and DEMON (marked ‡) are overlapping community detection baselines [45,50].

Event	Method	*K*	NMI(C;X)	*Q*	NMI(ref)	P	R	F1
R1	Louvain-Aggregate †	2	0.974	0.431	0.999	0.000	0.000	0.000
R1	SingleLayer-Vote	2	0.973	0.450	0.997	0.119	0.318	0.173
R1	Hard-Partition †	2	0.974	0.424	0.999	0.000	0.000	0.000
R1	Infomap †	12	0.970	0.439	0.995	0.000	0.000	0.000
R1	Spectral †	2	0.974	0.441	0.999	0.000	0.000	0.000
R1	SLPA ‡	2	0.973	0.435	0.998	0.280	0.227	0.251
R1	DEMON ‡	45	0.005	0.002	0.005	0.111	0.999	0.200
R1	Proposed	2	0.974	0.440	0.999	0.897	0.963	0.929
R2	Louvain-Aggregate †	2	0.806	0.411	0.998	0.000	0.000	0.000
R2	SingleLayer-Vote	2	0.804	0.387	0.994	0.349	0.497	0.410
R2	Hard-Partition †	2	0.806	0.403	0.998	0.000	0.000	0.000
R2	Infomap †	66	0.796	0.419	0.986	0.000	0.000	0.000
R2	Spectral †	2	0.806	0.402	0.999	0.000	0.000	0.000
R2	SLPA ‡	2	0.804	0.402	0.994	0.560	0.470	0.511
R2	DEMON ‡	68	0.008	0.003	0.007	0.162	0.970	0.278
R2	Proposed	2	0.806	0.420	0.998	0.700	0.973	0.814
R3	Louvain-Aggregate †	3	0.284	0.543	0.994	0.000	0.000	0.000
R3	SingleLayer-Vote	7	0.283	0.546	0.990	0.200	0.318	0.245
R3	Hard-Partition †	3	0.284	0.536	0.994	0.000	0.000	0.000
R3	Infomap †	39	0.283	0.549	0.989	0.000	0.000	0.000
R3	Spectral †	3	0.285	0.521	0.998	0.000	0.000	0.000
R3	SLPA ‡	2	0.283	0.535	0.993	0.350	0.295	0.320
R3	DEMON ‡	82	0.012	0.006	0.010	0.420	0.760	0.541
R3	Proposed	3	0.284	0.538	0.994	0.746	0.728	0.737

**Table 5:** Betweenness centrality of bridging and non-bridging users on the repost layer. The rank-biserial correlation *r* is the median over 50 subsampled comparisons of 500 users per group.

Event	$N_{\mathrm{bridge}}$	Fraction	Median BC (Bridge)	Median BC (Non-Bridge)	*r*
R1	2151	0.119	307.2	92.3	0.287
R2	11,012	0.198	129.6	94.2	0.078
R3	13,670	0.195	141.6	81.6	0.144

**Table 6:** Sensitivity of the co-opetition quantities to the sentiment rule for reposts without added text. The last column states whether all diagonal entries of the co-opetition matrix are positive.

Event	Rule	Cooperation Rate	Competition Rate	Diagonal Positive
R1	default positive	0.96	0.65	yes
R1	exclude	0.95	0.72	yes
R1	default negative	0.82	0.74	yes
R2	default positive	0.96	0.57	yes
R2	exclude	0.95	0.62	yes
R2	default negative	0.81	0.66	yes
R3	default positive	0.95	0.38	yes
R3	exclude	0.94	0.42	yes
R3	default negative	0.81	0.48	yes

## Data Availability

The complete analysis code is deposited at Zenodo (https://doi.org/10.5281/zenodo.22127291). Anonymized edge lists and derived labels are available at the same repository. Raw text and user identifiers are not shared due to platform policy restrictions but are available from the corresponding author on request.

## Abbreviations

The following abbreviations are used in this manuscript:

NMI	Normalized Mutual Information
BIC	Bayesian Information Criterion
BC	Bimodality Coefficient and Betweenness Centrality, as indicated in the text
